# Clinical and immunopathological findings during long term follow-up in *Leishmania infantum* experimentally infected dogs

**DOI:** 10.1038/s41598-017-15651-8

**Published:** 2017-11-21

**Authors:** Melissa Moura Costa Abbehusen, Valter dos Anjos Almeida, Manuela da S. Solcà, Laís da Silva Pereira, Dirceu Joaquim Costa, Leonardo Gil-Santana, Patricia Torres Bozza, Deborah Bittencourt Moté Fraga, Patrícia Sampaio Tavares Veras, Washington Luis Conrado dos-Santos, Bruno Bezerril Andrade, Claudia Ida Brodskyn

**Affiliations:** 1 Instituto Gonçalo Moniz, Fundação Oswaldo Cruz, Salvador, Bahia, Brazil; 2 Universidade Estadual de Vitória da Conquista, Vitória da Conquista, Bahia, Brazil; 3 Laboratório de Imunofarmacologia, Instituto Oswaldo Cruz, Biomanguinhos, Rio de janeiro, Rio de Janeiro, Brazil; 4 Departamento de Medicina Veterinária Preventiva e Produção Animal, Escola de Medicina Veterinária e Zootecnia, Universidade Federal da Bahia, Salvador, Bahia, Brazil; 5 Instituto de Ciência e Tecnologia de Doenças Tropicais, INCT-DT, Bahia, Brazil; 6 Multinational Organization Network Sponsoring Translational and Epidemiological Research (MONSTER) Initiative, Fundação José Silveira, Salvador, Bahia, Brazil; 7 Escola Bahiana de Medicina e Saúde Pública, Salvador, Bahia, Brazil; 8 Universidade Salvador (UNIFACS), Laureate Universities, Salvador, Bahia, Brazil; 9 Instituto de Ciências da Saúde, Universidade Federal da Bahia, Salvador, Bahia, Brazil; 10 Instituto de Investigação em Imunologia, São Paulo, São Paulo, Brazil

## Abstract

Canine Visceral Leishmaniasis (CVL) is caused by *Leishmania infantum*, which in the New World is transmitted by *Lutzomyia longipalpis*. While prospective clinical and immunological assessments of dogs experimentally challenged with *L. infantum* have been previously reported over a relatively short follow-up period, the long-term characterization of infected animals has not been performed to date. We evaluated dogs in a subclinical state for six years following experimental infection with *L. infantum* and *Lu. longipalpis* saliva, via an intradermal route, to characterize clinical, parasitological and immunological parameters arising from *L. infantum* experimental infection. We also assess these parameters in a group of naturally infected animals. The immune profiles of the experimentally and naturally infected animals exhibited increases of IFN-γ, IL-6 and IL-18, and decreases in TNF, IL-2, IL-8 and CXCL1, compared to controls. Our results indicate that over a six-year follow-up post-challenge, subclinically infected dogs presented low CVL clinical scores despite the persistence of *Leishmania* parasites in the lymph nodes, spleen and skin. Similarities observed among immune profiles in the context of experimental and natural infection seem to suggest that an enduring activation of the host immune response may lead to the control of parasite growth, thereby limiting disease severity.

## Introduction

Visceral leishmaniasis (VL) arising from *L. infantum* is a severe, often fatal, zoonotic disease, which represents one of the most relevant and challenging emerging diseases worldwide. The number of annual cases detected in Brazil comprises approximately 90% of the human cases occurring in Latin America^1,2^.

Dogs are considered the main domestic reservoir of the etiological agent of VL, *L. infantum*, and canine cases often precede the occurrence of human cases due the close proximity between dogs and humans^2–4^. This protozoan is mainly transmitted through the bite of infected sand flies, namely *Lu. longipalpis* in Brazil. The course of infection is highly variable, as not all infected dogs eventually present clinical signs of disease^5^.

The pathogenesis of CVL is highly associated with the immune response elicited against parasites by an infected dog^6^. The resistance profile is associated with the development of a specific anti-*Leishmania* cell-mediated response, resulting in the production of proinflammatory cytokines, such as IFN-γ and TNF, which increase the leishmanicidal activity of macrophages through the production of nitric oxide (NO) and reactive oxygen species (ROS)^7,8^. A susceptible profile is associated with parasite dissemination and exacerbated proliferation in combination with elevated antibody levels and a suppressed cellular immune response^7,9^.

CVL is a systemic disease with variable clinical signs, including lymphadenopathy, splenomegaly, weight loss, onycogryphosis and skin alterations^10^. Alterations in laboratory parameters, particularly hematological, often reveal normocytic anemia, thrombocytopenia, as well as mild or exacerbated leucopenia or lymphocytosis. Biochemical alterations, including hepatic and renal failure, can also be present^11^. Hypergammaglobulinemia is one of the most common findings, resulting in immune complex deposition and activation of the complement system, which may lead to glomerulonephritis and renal failure^10,12^. Typical histopathological findings in infected tissues consist of granulomatous inflammatory reactions associated with the presence of *Leishmania* amastigotes within macrophages^13^, in addition to disorganized splenic tissue^14^.

Our group designed an experimental challenge model of CVL that employed a combination of *Leishmania* and sand fly saliva. In this model, challenged animals exhibited similarities in clinical signs, parasite load and cytokine profiles when compared with naturally infected dogs^15^. In a previous study, only 34% (12/35) of the studied dogs remained in a subclinical state, displaying few clinical signs. Upon conclusion of the study period, the 12 subclinical dogs were maintained in the kennel for an additional four years, representing a total follow-up period of six years post-infection. These animals were clinically monitored at least twice annually throughout the evaluation period, during which time no change in clinical status was observed.

The present study details the evaluation of clinical and pathological parameters in these experimentally infected dogs for a follow-up period lasting for six years after infection. We found a persistence of parasites in the lymph nodes, spleen and skin, as well as a mild inflammatory profile that possibly allowed for infection control. Alterations in histopathological findings provide further evidence regarding the persistence of parasites, despite the fact that these dogs maintained a subclinical state. Importantly, similar results were also found in naturally infected dogs that had comparable clinical scores, suggesting that the asymptomatic state could persist for longer periods than what has been previously reported^16,17^.

## Results

All presented parameters were obtained from canine tissue samples at the time of euthanization, six years after infection. Laboratory results of these dogs 450 days post-infection are listed in Supplementary Table S3. Tables 1, 2 and 3 delineate the clinical and clinicopathological findings of the euthanized animals. Considering all the results evaluated at regular intervals throughout the 6-year follow-up period, no significant changes were detected in the observed parameters.

**Table 1:** VL clinical parameters employed to calculate the clinical score of each animal included in the study.

Parameters	Score
Nutritional status	0–2
Alopecia	0–2
Onychogryphosis	0–2
Mucosal color	0–2
Splenomegaly	0–2
Lymphadenopathy	0–2
Conjunctivitis	0–2
Mucosal Lesion	0–2
**Total Score**	**16**

**Table 2:** Clinical signs observed in experimentally infected dogs by *Leishmania infantum* 6 years after infection.

Clinical Sign	Number	Percentage
Nutritional status	1	8%
Alopecia	3	25%
Onychogryphosis	0	0
Mucosal color	0	0
Splenomegaly	0	0
Lymphadenopathy	6	50%
Conjunctivitis	3	25%
More than 1 clinical sign	4	33%

**Table 3:** Haemathological and Biochemical Findings observed in experimentally infected dogs by *Leishmania infantum* 6 years after infection.

Haematological Findings	Number of Dogs	%
Low hemoglobin	12	50%
Low hematocrit	08	67%
Leukopenia	6	25%
Lymphopenia	4	33
Thrombocytopenia	2	17%
**Biochemical Findings**	**Number of Dogs**	**%**
High Urea	08	67%
ALT altered levels	06	50%
AST altered levels	04	33%

### Clinical scores from dogs six years after experimental infection

Thirty-five dogs were infected at the outset of the study. After infection and during follow-up, only 34% (n = 12) maintained a subclinical state. Clinical assessments of these dogs were performed by evaluating the severity of clinical manifestations arising from *L. infantum* infection, as characterized by weight loss, focal or generalized dermatitis, mucous membrane lesions, onychogryphosis, splenomegaly, lymphadenopathy, conjunctivitis and pale mucous membranes. Six years after experimental infection, the most frequently observed clinical signs were lymphadenopathy (50%), skin pathologies and conjunctivitis (25%) (Table 1). Other clinical signals typically observed in *Leishmania*-infected diseased dogs, such as onychogryphosis, pale mucosal and cachexia, were absent in the studied animals, indicating the development of a mild form of disease (Table 2).

### Hematological and Biochemical Evaluation

Hematological and biochemical parameters were assessed to complement clinical evaluations when dogs were euthanized six years after infection. In the erythrocyte series, the majority of subclinical dogs exhibited a normocytic normochromic anemia profile, with 6 of 12 (50%) presenting hemoglobin levels below standard values and 8 (67%) had decreased levels of erythrocytes and hematocrit. In a white cell panel, 3 of 12 (25%) presented leukopenia and four presented (33%) lymphopenia. Despite thrombocytopenia being a common finding in CVL, this was found in just two animals (17%) (Table 3 and Supplementary Table S1).

The kidney profile revealed that while all animals had normal levels of creatinine, 8 of 12 (67%) subclinical dogs showed levels of urea above standard values. Additionally, the liver profile of these animals showed altered levels of ALT (6/12–50%) and AST (4/12–33%). Although increases in total protein and globulin, as well as decreases in albumin, are important features when diagnosing CVL, none of the evaluated dogs presented significant alterations in these parameters (Supplementary Table S2).

### Histopathology

**Figure 1:**
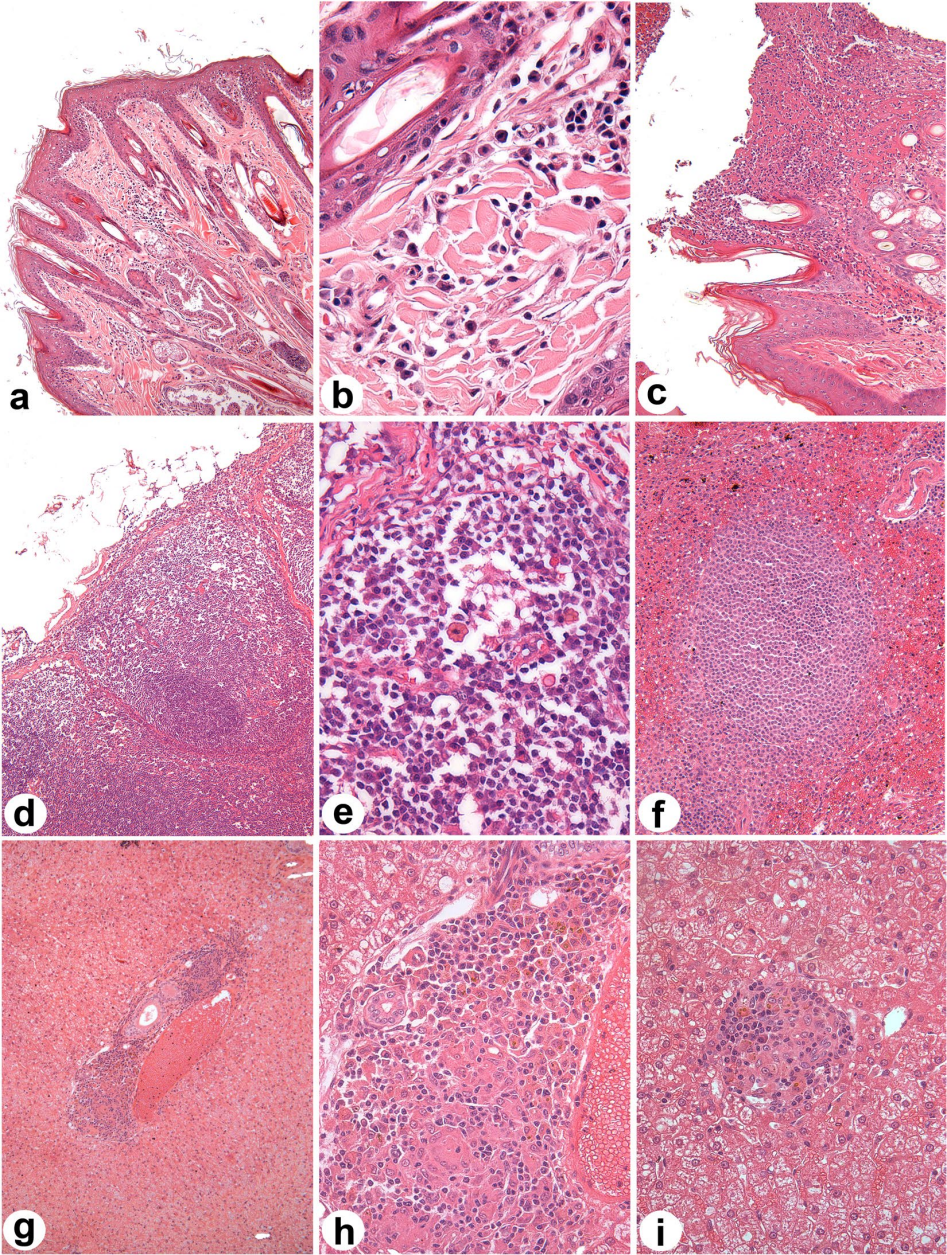
Histopatological aspects in, skin, lymph node, spleen and liver of experimentally infected dogs, six years after challenge. Dog were intradermally infected with 10^7^ parasites plus five pairs of *Lutzomyia longipalpis* salivary glands according the parameters described in Material and Methods section. Six years after infection, dogs were euthanized and different organ samples were collected, fixed and stained with H&E for histological evaluation. (**a**,**b**) Mononuclear inflammatory infiltrate in the skin; (**c**) skin ulcer displaying neutrophils and fibrin; (**d**) atrophic lymphoid follicles in popliteal lymph nodes; (**e**) plasmocytosis and erythrophagocytosis in popliteal lymph node; (**f**) disorganized splenic white pulp in spleen; (**g**) periportal inflammatory infiltrate; (**h**,**i**) granuloma in liver sections from experimentally infected dogs.

For histological analysis, samples were collected six years after infection from canine skin, popliteal lymph node, liver and spleen tissue (Fig. 1). In the ears of *Leishmania*-infected dogs, at a location other than the inoculation site, some mild lymphoplasmacytic pleomorphic chronic inflammatory infiltrate was present in 83% (10/12) of the infected animals (Fig. 1a and b). In a single dog, it was possible to observe an ulcer with neutrophils and fibrin at the most superficial layers of the dermis (Fig. 1c). Popliteal lymph nodes presented atrophic lymphoid follicles in 42% (5/12) of the animals (Fig. 1d), while 58% (7/12) had normal reactive lymphoid follicles (Data not shown). Plasmacytosis in 83% (10/12) and erythrophagocytosis in 67% (8/12) were also frequently observed in the popliteal lymph nodes of *Leishmania*-infected animals (Fig. 1e). Splenic white pulp disorganization was seen in 25% (3/12) (Fig. 1f). The liver presented periportal inflammatory infiltrate (Fig. 1g) and granulomas in portal spaces and the parenchyma in 67% (8/12) (Fig. 1h and i) of the *Leishmania*-infected dogs.

### Clinical, Serological and Parasitological Parameters

All dogs underwent clinical evaluations prior to and after challenge with *L. infantum* in the presence of sand fly saliva. In these evaluations, clinical assessments were performed via the evaluation of CVL signs. We observed that 83% (10/12) of the animals presented a clinical score <2 (Table 4), while only 17% (2/12) displayed clinical scores of 4 or 5, which reinforces their subclinical status. No animals showed any differences in clinical scoring throughout the 6-year follow-up period (data not shown).

**Table 4:** Serological and Parasitological parameters of experimentally infected dogs at 450 days, 6 years after infection and naturally infected dogs.

EXPERIMENTALLY INFECTED DOGS					
450 DAYS					
Samples	Clinical Score	DPP (rK28)	Parasite Load Lymph Node (1 mg DNA)	Parasite Load Spleen (1 mg DNA)	Parasite Load Skin (1 mg DNA)
BMG 01	5	P	4120	ND	ND
BMG 02	2	N	700	ND	ND
BMG 03	3	P	4120	ND	ND
BMG 04	2	N	10640	ND	ND
BMG 05	5	N	12040	ND	ND
BMG 06	2	N	24740	ND	ND
BMG 07	1	N	12200	ND	ND
BMG 08	4	N	5380	ND	ND
BMG 09	0	P	22560	ND	ND
BMG 10	0	P	9540	ND	ND
BMG 11	1	P	9620	ND	ND
BMG 12	0	P	10220	ND	ND
**TOTAL**	**—**	**50% (6/12)**	**100% (12/12)**	**—**	**—**
**6 YEARS**					
BMG 01	5	N	30.9	0	0
BMG 02	0	N	295.1	1.5	0
BMG 03	4	P	540.1	67.6	0
BMG 04	0	N	12,3	0.	0
BMG 05	2	N	419.4	0	0
BMG 06	1	N	4.3	1.5	0
BMG 07	0	P	4050.2	9.9	0
BMG 08	1	P	1.6	0	3.1
BMG 09	2	P	61354	140.2	4.8
BMG 10	1	P	7078.2	0	94.6
BMG 11	1	P	1.4	0.	120.5
BMG 12	0	P	2.8	0	1228.3
**TOTAL**	**—**	**58% (7/12)**	**100% (12/12)**	**42% (5/12)**	**42% (5/12)**
**NATURALLY INFECTED DOGS**					
RUR 093	2	P	ND	181462,0	2194,0
RUR 127	1	P	ND	38,9	0,0
RUR 044	2	P	ND	325956,6	76,2
RUR 060	2	P	ND	1103728,9	0,0
RUR 168	0	P	ND	20286,7	3224,7
RUR 158	3	P	ND	36068,1	429,5
RUR 160	2	P	ND	9442,0	432,3
RUR 169	0	P	ND	9,3	1,4
RUR 012	3	P	ND	2946,0	78,8
RUR 065	1	P	ND	155257,0	3958,4
RUR 094	2	P	ND	163,9	0,0
RUR 141	2	P	ND	233,7	752,6
**TOTAL**	**—**	**100% (12/12)**	**—**	**100% (12/12)**	**75% (9/12)**

Our analysis of anti-k28 antibodies showed that 58% (7/12) of the dogs had detectable levels of IgG anti-*Leishmania* (Table 4). All dogs presented converted IgG anti-SLA levels 90 days after the challenge (data not shown).

Spleen, popliteal lymph node and skin tissue biopsies were performed to quantify parasite load by qPCR using primers specific to *L. infantum*. Parasites were detected in 100% (12/12) of the lymph nodes and 58.3% (7/12) of the spleens and skin. Additionally, a single dog presented parasites in all evaluated organs. All animals had already presented parasites in the lymph nodes at 450 days after challenge, which persisted until the time of euthanization (Table 4). As shown in Table 4, naturally infected dogs had similar clinical scores (0–3), and all presented IgG anti-k28 in addition to parasites in the spleen, whereas 75% (9/12) had *Leishmania* in the skin. Parasite loads in this group of animals were higher than those in experimentally infected dogs, which could be explained by differences in infection duration, as well as exposure to different living conditions, e.g. malnutrition or co-infections (Babesia and Erlichia), as was previously described in the literature as aggravating factors of CVL^18^.

### Cytokine and Chemokine evaluation

**Figure 2:**
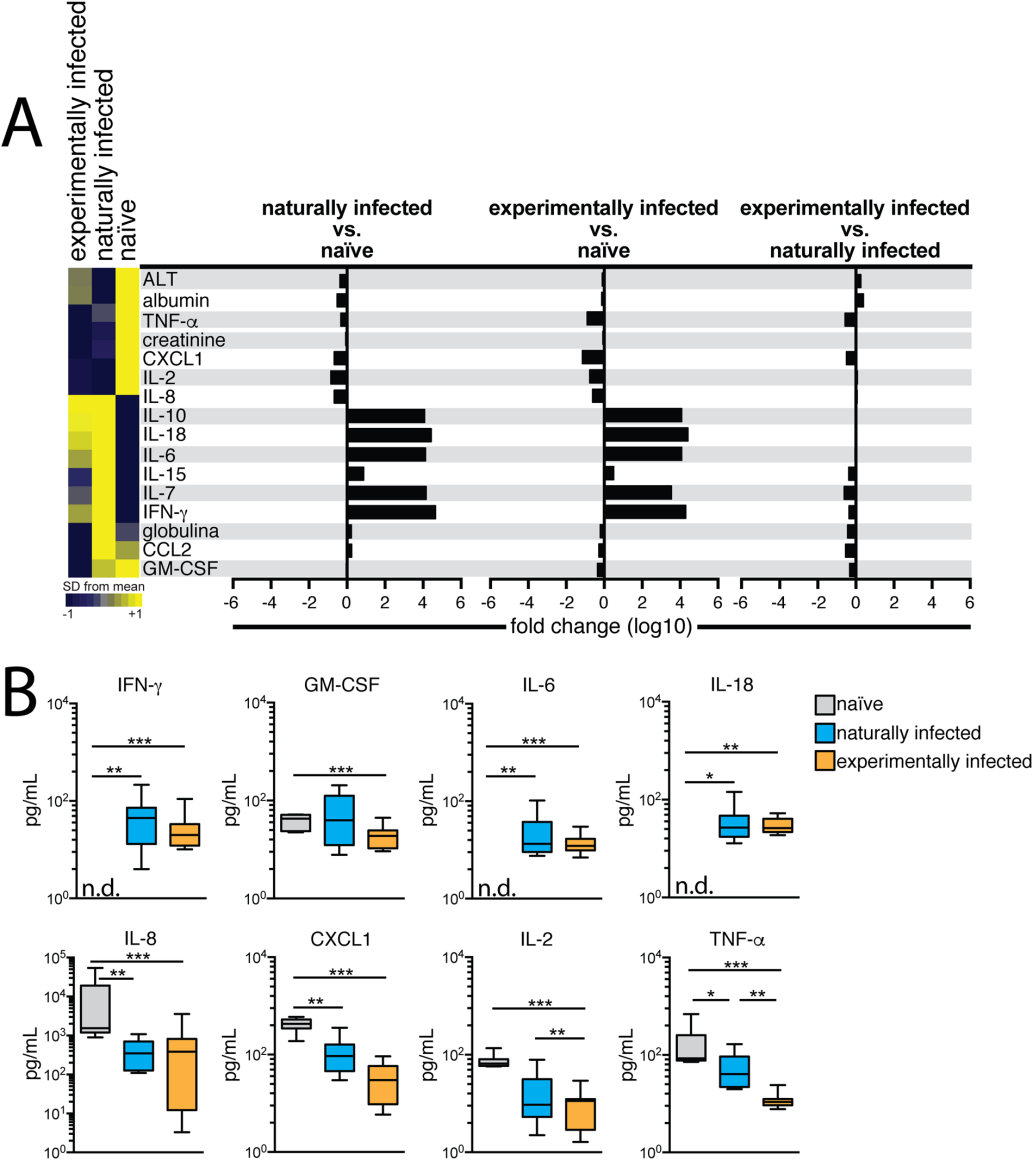
Cytokines and chemokines detected in sera of experimentally infected dogs, six years after challenge. Dogs were intradermally infected with 10^7^ parasites plus five pairs of *Lutzomyia longipalpis* salivary glands according the parameters described in Material and Methods section. Six years after infection, dogs were euthanized and sera were collected for Luminex evaluation. (**A**) Hierarchical cluster analysis and Spearman correlations was performed to depict the overall expression profile of the indicated serum biomarkers in the different study groups. (**B**) Serum biomarkers presented by naiive, naturally and experimentally infected dogs IFN-γ, GM-CSF, IL-6, IL-8, IL-18, CXCL1, IL-2, TNF.

Inflammatory cytokines and chemokines were evaluated in the sera of non-infected naïve, naturally and experimentally subclinically infected dogs. To compare differences in these parameters, we performed hierarchical cluster analysis with bootstrapping to depict the overall expression profile of the indicated serum markers among the different study groups. We found that both groups of infected dogs showed similar signatures, presenting lower concentrations of ALT, albumin, TNF, creatinine, CXCL1, IL-2 and IL-8, as well as higher levels of, IL-10, IL-18, IL-6 and IFN-γ in comparison to naïve dogs. On the other hand, lower concentrations of IL-15, IL-7 and globulin were seen in naïve and experimentally infected dogs compared to naturally infected dogs. Naïve and naturally infected dogs showed similar signatures concerning CCL2 and GM-CSF expression (Fig. 2A).

As shown in Fig. 2B, both naturally and experimentally infected groups exhibited significantly higher levels of IFN-γ, IL-6 and IL-18 compared to the naïve group (p < 0.001). However, the infected (experimentally and naturally) groups presented lower IL-8, CXCL1, IL-2 and TNF levels in comparison to naïve animals (p < 0.001). Experimentally infected dogs presented a significant decrease in GM-CSF compared to controls (p < 0.001). Although other cytokines and chemokines were also evaluated, i.e. IL-7, IL-15 and CXCL-10, no significant differences were detected among the naïve and infected dogs (Data not shown).

## Discussion

In long-term prospective studies conducted in areas endemic for CLV, a limited percentage of naturally infected dogs are observed to become very ill, while the vast majority of animals seem to remain healthy for many years^19–21^. In areas endemic for CVL in Brazil, the prevalence of symptomatic dogs varies from 30 to 50%, i.e. a high proportion of dogs remain in asymptomatic state. In nature, dogs are commonly exposed to a variety of adversities, and in a given endemic area it is possible that all have similar chances to develop symptomatic disease following infection with *L. infantum*. There is a consensus in the literature that the immune response elicited against *Leishmania* is essential to the outcome of infection^3,22,23^. Different routes have been employed to experimentally infect dogs, and limitations associated with intradermal, endovenous or subcutaneous routes of injection have been discussed in the literature^24–26^. An ideal route would be to employ infected sand flies, as reported by the Shaden Kamhawi research group^24^. Unfortunately, this type of experimentation requires a robust insect colony, which few laboratories possess. In an attempt to mimic the conditions surrounding natural infection, we chose to infect the presently studied dogs intradermally using *Leishmania* and *Lu. longipalpis* saliva^15,22,25^. In addition, sand fly saliva possesses potentially immunomodulatory properties that favor the establishment of infection^27,28^. A previous study conducted by our group employing an intradermal route of infection found the presence of saliva throughout a 450-day follow-up period^15^.

We infected 35 dogs and 23 were euthanized after 450 days upon the presentation of severe disease, with clinical scores >8. Twelve dogs remained asymptomatic, and these animals were followed-up throughout the course of the present study. These dogs showed few clinical signs related to CVL. The most frequent were lymphadenomegaly (50%), followed by skin lesions and conjunctivitis (25%). These clinical signs have been extensively described in the literature in naturally infected dogs^29–32^, notably lymph node enlargement (mostly the popliteal and pharynx). Interestingly, all variable numbers of parasites were found in the lymph nodes of the experimentally infected dogs, which could contribute to enlargement. In addition, skin abnormalities are often relevantly characterized by dry seborrheic dermatitis, desquamation and associated alopecia, since diseased dogs presenting these skin alterations seem to be associated with the ability to transmit *L. infantum* to the sand fly population^31,33^. On the other hand, Solano-Gallego *et al*. (2004) found similar parasite loads in affected skin as well as in the normal, healthy skin of infected dogs^34^. Indeed, the present study found just three dogs presenting alopecia, while qPCR detected the presence of parasites in the healthy skin of five dogs (42%). Moreover, parasites could also be present in different areas of healthy skin, suggesting the role of subclinical dogs as transmitters of parasites to uninfected sand flies^24^.

Despite alterations in hematological parameters and the biochemical profiles of dogs naturally infected by *L. infantum*, these characteristics are of limited use in disease diagnosis. Still, they may represent important biomarkers for evaluating the clinical progress of infected animals, thereby contributing to a better understanding of CVL pathogenesis^35^. In dogs with active CVL disease, anemia has been associated with a disorder in the erythroid bone marrow compartment^36^. This could also be related to increased hemolysis, due the trapping of erythrocytes in the enlarged spleen and liver in association with an inflammatory response to *L. infantum* infection^37^.

In a white cell panel, 25% of the evaluated dogs presented leukopenia and 33% presented lymphopenia, yet only 17% had thrombocytopenia. Similar results were observed by Reis *et al*.^36^ and Nicolato *et al*.^17^ in naturally infected dogs. The low mean platelet counts detected herein is a very common laboratorial sign in CVL, and could be associated to vasculitis or platelet destruction following renal and/or hepatic failure, or anti-platelet antibodies^38,39^. However, in our study, we found thrombocytopenia in just two dogs, which reinforces the notion that dogs evaluated over the long-term present similar pathological clinical profiles as those naturally exposed to *Leishmania* parasites that course with a subclinical status.

Biochemical parameter evaluation allowed us to evaluate liver and kidney profiles by quantifying AST, ALT, urea, creatinine and total proteins and fractions. We observed that 67% of the asymptomatic animals showed higher levels of urea with no alterations in creatinine, while only 50% presented slightly higher levels of ALT versus 33% of AST, which denotes mild dysfunction of the liver and kidney. We can hypothesize that these alterations may reflect the onset of renal injury, since changes in creatinine only occur in response to severe renal dysfunction. According to Baneth *et al*.^32^, azotemia becomes evident only when the majority of nephrons are dysfunctional, which occurs rather late in disease progression.

Histopathological analysis detected an inflammatory profile characterized by intrasinusoidal leukocytosis in all infected dogs, with 67% of the animals presenting granulomas in the liver. According to Tafuri *et al*.^40^, granulomas are the most significant lesion type observed in the livers of *L. infantum*-infected dogs, which are characterized by intralobular hepatic granulomatous formations with varying numbers of macrophages. Moreover, these liver granulomas could be indicative of an active cell-mediated immune response, as was observed in resistant dogs naturally infected in endemic areas^41^. These findings could suggest that the experimentally infected dogs developed similar hepatic alterations as those presented by naturally resistant infected dogs.

To better understand the immune response presented by the studied dogs six years after infection, hierarchical cluster analysis was performed to obtain biosignatures regarding the serum parameters evaluated. In the heatmap shown in Fig. 2A, both experimentally and naturally infected dogs exhibited similar profiles regarding lower levels of TNF, CXCL1, IL-2 and IL-8 when compared to naïve dogs. Moreover, in Fig. 2B, statistical differences among the naturally and experimentally infected dogs were see with respect to levels of IL-2 and TNF, which indicates that these cytokines present distinct behavior in accordance with different courses of infection. It is important to note that these features could also be attributed to co-infections, such as babesiosis or erlichiosis, which have been very commonly reported in our area of study^18,42^.

A similar profile was observed with regard to GM-CSF levels, in which experimentally infected groups showed a significant decrease in this cytokine compared to the control group.

A recent report from our group evaluating serum biomarkers characteristic of CVL severity found similar results to those shown herein, i.e. decreases in these mediators in subclinical naturally infected animals, which serves to validate our animal infection model^42^. Infected groups had higher expression of IL-18, IL-6 and IL-10 compared to naïve animals, which suggests a mixture of inflammatory and anti-inflammatory responses. Serum levels of IL-7 and IL15 were higher in naturally infected dogs compared to naïve and experimentally infected dogs, yet this was not statistically significant. By contrast, IFN-γ levels among the experimentally and naturally infected dogs were statistically higher in comparison to naïve dogs, which denotes an immune response capable of controlling parasite proliferation and dissemination.

Concerning CXCL-1, a chemokine responsible for the recruitment of neutrophils^43^, we noted that subclinically infected animals had decreased levels in comparison to the naïve group. In a study previously performed by our group, we found a significant increase in this chemokine in diseased naturally infected dogs^42^. Accordingly, we can infer that low parasite loads could regulate the production of CXCL1, which may lead to a milder inflammatory cellular immune response.

Our analysis of lymph node parasite load and the humoral immune response against a *Leishmania* antigen (rK28) found that although all dogs presented parasites in this organ, only 58% presented detectable levels of IgG anti-*Leishmania*. This seems to suggest that this serological test is not appropriate for CVL diagnosis among subclinical infected dogs^44^, while PCR for the detection of parasites is more accurate. Our collaborators on a study performed in an endemic area described similar findings. Interestingly, 100% of those dogs tested positive in at least one of the tissue samples analyzed using qPCR^45^. Indeed, there seems to be a strong correlation between lymph node immune response and the control of clinical status during ongoing CVL. Giunchetti *et al*.^46^ found an intense hypertrophy/hyperplasia in the lymph nodes of subclinically infected dogs, suggesting that lymphocyte activation in the lymph nodes may favor cell migration and control of parasite burden in parasitized organs, yet they could not find any relationship to clinical CVL status^46^.

Considering the varying degrees of infection and clinical manifestations observed in the skin and lymphoid tissues of the animals followed for six years after being intradermally challenged with *L. infantum* in the presence of *L. longipalpis* saliva, we can infer that these dogs exhibited similar clinical, immunological and pathological profiles as those that were naturally infected and maintained a subclinical status. There is a debate in the literature regarding the role of subclinically infected dogs with respect to the maintenance of infection. Our results demonstrated the presence of parasites in the skin, even in areas where no lesions were apparent, in at least in 41% of the animals evaluated. This feature calls attention to the potential ability of these animals to transmit parasites to uninfected sand flies.

In sum, the present study showed that experimentally infected dogs that were well-nourished, free of ectoparasites and worms, and without any co-infections, nonetheless exhibited an immune response characteristic of CVL, together with clinical and pathological findings resembling those observed in naturally infected animals. It is our hope that these findings may further the understanding of the subclinical status of this disease, which could play a role in controlling *Leishmania* growth in addition to the spread of parasites to the visceral organs.

## Methods

### Animals

Thirty-five healthy, 3-month-old beagle dogs of both genders were purchased from a local breeder in a non-endemic area from Brazil (Tad’s Henriques Kennel, Colombo, Paraná State, Brazil). After quarantine, all dogs received routine vaccinations (rabies, distemper, hepatitis/adenovirus type2, leptospirosis and parvovirus) and were dewormed against helminthes. Prior to challenge, blood samples were collected for serological evaluations and no dogs showed any detectable levels of anti-*Leishmania* or anti-saliva (*Lu. longipalpis*) antibodies. Throughout the study, the animals were housed at an experimentation kennel located in the municipality of Monte Gordo, Bahia State, Brazil facility that possessed an infrastructure suited to safely handle infected animals. Only 34% (12/35) of these dogs presented a subclinical state after infection and maintained this clinical profile during the 6-year follow-up period. The data presented in this manuscript refers to these 12 dogs.

Additionally, eight subclinical naturally infected mongrel dogs of both genders and different ages were obtained from a cross-sectional study conducted in the municipality of Camaçari, an area highly endemic for CVL. These dogs presented anti-*Leishmania* antibodies (DPP® CVL, Bio-Manguinhos Unit, Rio de Janeiro, Brazil) and positivity for *Leishmania* on qPCR. For the Luminex assays, we included sera from eight adult, non-infected (naïve) beagle dogs of both genders from a non-endemic CVL area, which tested negative for anti-*Leishmania* and anti-saliva antibodies (non-exposed dogs).

### Ethical Statement

All procedures performed herein were conducted in accordance with the guidelines for animal research established by the Brazilian College of Animal experimentation (Colégio Brasileiro de Experimentação Animal) and the National Council for Animal Control Experimentation (Conselho Nacional de Controle de Experimentação Animal). The IGM - FIOCRUZ Institutional Review Board for Animal Experimentation approved all procedures involving animals (CEUA – Instituto Gonçalo Muniz - IGM/FIOCRUZ - 010/2009).

### Sand Flies and SGH preparation

*L. longipalpis*, Cavunge strain (Cavunge, Bahia), were reared at the Laboratory of Imunoparasitology (LIP-IGM) as previously described^47^. Salivary glands were dissected from 5–7-day old females and stored in saline at −70 °C. Before use, salivary glands were sonicated and centrifuged at 8.000xg for 5 min. The supernatant was collected and used immediately.

### *Leishmania* parasites and intradermal experimental infection

For experimental infection, we employed the a previously described protocol^15^. Briefly, *L. infantum* (MCAN/BR/00/BA262) promastigotes, originally isolated from a naturally infected dog (Bahia State, Brazil), were obtained from an existing collection and cultured in Schneider’s medium (LGC, Brazil) supplemented with 10% heat inactivated FBS (fetal bovine serum), 2 mM L-glutamine, 100 IU/ml penicillin and 1% streptomycin. Dogs were intradermally inoculated in the ear with 10^7^ stationary-phase promastigotes in the presence of SGH equivalent to five pairs of glands using a 29-gauge needle at a volume of 200 µl. After infection, all dogs were housed in kennels covered with anti-insect netting, received a balanced diet and water *ad-libitum*. Once a year all animals were dewormed and received routine vaccinations.

### Clinical evaluation

During the first two years of study, all dogs were clinically evaluated on a monthly basis for CVL signs. In subsequent years, the animals were examined at 6-month intervals. The severity of CVL was determined by the presence or absence of the following clinical signs: nutritional status as represented by weight loss, mucosal color, skin involvement (alopecia), lymphadenopathy, splenomegaly, conjunctivitis, nail size (onychogryphosis) and the presence of mucosal lesions graded from 0 to 2 at each time point, as adapted from Manna *et al*.^48^. After each evaluation, as these points were summed and the corresponding value was considered as the clinical score of each dog (minimum score = 0; maximum score = 16).

### Euthanasia

Six years after infection, all dogs were euthanized by intravenous injection of an acepromazin/ketamin anesthetic prior to the administration of a supersaturated solution of potassium chloride. Death was confirmed by the presence of cardiac respiratory arrest.

### Hematological and Biochemical Parameters

Hematological and biochemical parameters were evaluated during the course of infection (each two months) until 450 days and in the day of the necropsy 6 years later. Total red blood cell and white blood cell counts were determined using an automated cell counter (Pentra 80 counter, ABX Diagnostics, Montpellier, France). Micro-hematocrit tubes containing blood samples were centrifuged at 12,000 rpm for 5 min, after which hematocrit levels were estimated. Serum was collected by centrifuging blood samples, then submitted to biochemical testing using an enzymatic colorimetric method with an A15 auto-analyzer (BioSystems, Barcelona, Spain) to evaluate total protein, globulin, albumin, AST (aspartine aminotransferase), ALT (alanine aminotransferase), urea and creatinine.

### Humoral immune response

Anti-*Leishmania* antibody levels were determined by the DPP CVL (Dual-Path Platform, Bio-Manguinhos) rapid test, which detects anti rk28-antibodies, as previously described^49^. All procedures were performed in accordance with manufacturer instructions.

### DNA extraction and parasite burden quantification by real-time PCR

Lymph node, skin and spleen fragment DNA was extracted using a DNeasy® Blood & Tissue Kit (Qiagen, Hilden, Germany) by following manufacturer protocols. After extraction, DNA quality and concentration was determined using a digital spectrophotometer (NanoDrop® ND-1000, Thermo Scientific, Wilmington, USA) and DNA integrity was evaluated on a 0.8% agarose gel. DNA samples were then adjusted to a concentration of 30 ng/µl and stored at −20 °C until the time of cPCR and qPCR assaying. To quantify *Leishmania* DNA in canine spleen fragments, qPCR assays were performed using a technique as previously described by Solcà *et al*.^45^. As a positive control, splenic aspirate samples from two dogs of an endemic area that tested positive for *Leishmania* infection were employed. Negative controls consisted of splenic aspirates from two healthy dogs from the municipality of Pelotas, Rio Grande do Sul, Brazil, a non-endemic area.

### Luminex Assay

Sera samples were profiled using a pre-defined Luminex-based multiparametric bead array kit (Milliplex Map Kit - canine cytokine magnetic bead panel, Life Technologies, Carlsbad, CA, USA) to measure the following canine inflammatory cytokines and chemokines: IFN-γ, IL-10, TNF, IL-1β, IL-2, IL-6, IL-7, IL-15, IL-8, MCP-1, CXCL-1 and GM-CSF. All procedures were performed in accordance with the manufacturer’s protocol. Briefly, a 96-well filter plate was blocked with washing buffer under agitation on a plate shaker at room temperature. Assay Buffer was then added with the appropriate matrix solution into the background, standard and control wells, followed by the addition of sera and premixed beads to each well. After incubation, the detection antibody was added followed by the conjugate (Streptavidine). After washing, results were read using a Luminex 200^TM^ and data were obtained using software by Luminex Corporation. Results are expressed in pg/mL and mean fluorescence intensity (MFI). These assays were performed at the Laboratório de Imunofarmacologia of the Oswaldo Cruz Institute, Fiocruz – RJ.

### Histological analysis

Slices 4-mm in thickness of liver, popliteal lymph node, spleen and skin tissue were fixed in formalin and embedded in paraffin. Hematoxylin-and eosin-stained sections with a thickness of 4 to 5 µm were examined by optical microscopy. Spleen samples were classified according to the degree of splenic white pulp organization using previously described criteria^13^.

### Statistical analysis

Statistical analysis was performed using GraphPad Prism 5.0 software (GraphPad Software, USA). Cytokine evaluations were performed using the non-parametric Mann Whitney U-test. Hierarchical cluster analysis (Ward’s method) with bootstrapping was performed to depict the overall expression profile of serum biomarkers in negative and naturally or experimentally infected dogs. Significant statistical differences among three or more groups were evaluated using the Kruskal-Wallis test with Dunn’s multiple comparisons. All analyses were two-tailed and pre-specified. Differences were considered significant when P values ≤ 0.05 after adjustment for multiple comparisons using the Holm-Bonferroni method.

## Electronic supplementary material

Supplementary Information
