# Supplementary material for: Clinical and immunopathological findings during long term follow-up in Leishmania infantum experimentally infected dogs
Source: Sci Rep. 2017 Nov 21;7:15914. doi: 10.1038/s41598-017-15651-8 (PMC5698407; doi:10.1038/s41598-017-15651-8)
Supplement: Supplementary file 1 — Supplementary Information [file 41598_2017_15651_MOESM1_ESM.pdf]

Clinical and immunopathological findings during long term follow-up in *Leishmania infantum* experimentally infected dogs

Melissa Moura Costa Abbehusen[1], Valter dos Anjos Almeida[1], Manuela da S Solcà[1], Laís da Silva Pereira[1], Dirceu Joaquim Costa[2], Leonardo Santana[1], Patricia Torres Bozza[3], Deborah Bittencourt Moté Fraga [1,4,5], Patrícia Sampaio Tavares Veras[1,5], Washington Luis Conrado dos-Santos[1], Bruno Bezerril Andrade[1,6,7,8], Claudia Ida Brodskyn[1,4,9,10,*]

[1]Instituto Gonçalo Moniz, Fundação Oswaldo Cruz, Salvador, Bahia, Brazil

[2]Universidade Estadual de Vitória da Conquista, Vitória da Conquista, Bahia, Brazil

[3] Laboratório de Imunofarmacologia, Instituto Oswaldo Cruz, Biomanguinhos, Rio de janeiro, Rio de Janeiro, Brazil

[4]Departamento de Medicina Veterinária Preventiva e Produção Animal, Escola de Medicina Veterinária e Zootecnia, Universidade Federal da Bahia, Salvador, Bahia, Brazil

[5]Instituto de Ciência e Tecnologia de Doenças Tropicais, INCT-DT, Bahia, Brazil

[6]Multinational Organization Network Sponsoring Translational and Epidemiological Research (MONSTER) Initiative, Fundação José Silveira, Salvador, Bahia, Brazil

[7]Escola Bahiana de Medicina e Saúde Pública, Salvador, Bahia, Brazil

[8]Universidade Salvador (UNIFACS), Laureate Universities, Salvador, Bahia, Brazil

[9]Instituto de Ciências da Saúde, Universidade Federal da Bahia, Salvador, Bahia, Brazil

[10]Instituto de Investigação em Imunologia, São Paulo, São Paulo, Brazil

Supplementary Table 1 - Haemathological Parameters observed in experimentally infected dogs by *Leishmania infantum* 6 years after infection

HAEMATOLOGICAL	Reference value	BMG 1	BMG 2	BMG 3	BMG 4	BMG 5	BMG 6	BMG 7	BMG 8	BMG 9	BMG 10	BMG 11	BMG 12
Erythrocytes (10^6/mm3)	5400-7800	5300	5900	5600	5000	6300	4800	4300	4800	4900	5100	6500	5100
Hemoglobin (g%)	13-19	12.98	13.80	13.50	12.00	14.00	10.80	11.40	12.80	12.00	13.40	16.00	13.10
Hematocrit (%)	37-54	35.6	39	39	34	41	31	30	34	32	35	42	35
Leucocytes (10^3/mm^3)	7000-14000	9563	8100	6700	7800	9300	10700	6600	12600	14900	9900	5699	12900
Segmented neutrophils	3600-11500	6860	4860	4690	6240	6975	7490	5280	10710	13410	7920	3920	10836
Eosinophils	100-1250	552	810	536	624	930	1070	264	630	149	297	112	645
Lymphocytes	1000-4800	1090	1782	1340	780	1023	1070	792	756	894	1287	1232	1032
Monocytes	150-1350	429	648	134	156	372	1070	264	504	447	396	336	387
Platelets (10^5/mm3)	160-430000	221333	88000	156000	309000	379000	365000	193000	275000	274000	160000	210000	247000

Suplementary Table 2 - Biochemical Parameters observed in experimentally infected dogs by *Leishmania infantum* 6 years after infection

BIOCHEMICAL	Reference Value	BMG 1	BMG 2	BMG 3	BMG 4	BMG 5	BMG 6	BMG 7	BMG 8	BMG 9	BMG 10	BMG 11	BMG 12
**Total Proteins**	5.5-7.5	6.8	7.2	7.1	5.8	6.3	5.7	6.8	6.5	6.7	6.6	6.2	5.8
**Albumin**	2.4-4.3	2.61		2.74	3.13	3.67	3.2	3.45	3.37	3.14	2.65	3.32	3.21
**Globulin**	2.7-4.4	3.16		2.64	1.52	1.81	1.54	1.83	1.52	2.13	2.73	1.67	1.62
**a/g**	0,89-0,97	0.62		0.62	1.17	1.39	1.28	1.03	1.07	0.87	0.66	1.15	1.24
**Creatinin**	0.7-1.6	1.1	0.9	0.8	0.7	0.7	0.6	0.9	0.7	0.8	0.8	1	0.8
**Ureia**	8.8-25.9	19	30	27	20	20	25	37	33	31	31	44	30
**ALT**	<50	12	179	41	41	57	28	558	31	42	72	72	144
**AST**	8.9-48.5	5	46	40	39	24	23	109	27	31	27	97	52

Suplementary Table 3 - Biochemical Parameters observed in experimentally infected dogs by *Leishmania infantum* at 450 days after infection

BIOCHEMICAL	Reference Value	BMG 1	BMG 2	BMG 3	BMG 4	BMG 5	BMG 6	BMG 7	BMG 8	BMG 9	BMG 10	BMG 11	BMG 12
**Total Proteins**	5.5-7.5	7,4	7,2	7,4	6,8	7	7,2	7	7,4	6,4	6,8	6,8	7,4
**Creatinin**	0.7-1.6	0,91	0,95	1,02	1,32	0,98	1,29	1,15	1,02	1,05	0,91	0,98	0,91
**ALT**	<50	22,17	13,49	13,49	22,17	6,74	13,97	9,15	8,19	13,49	14,46	11,56	13,49
